# Cytochrome *c* and cancer cell metabolism: A new perspective

**DOI:** 10.1016/j.jsps.2024.102194

**Published:** 2024-10-31

**Authors:** Bader Alshehri

**Affiliations:** Department of Medical Laboratory Sciences, College of Applied Medical Sciences, Majmaah University, Almajmaah-11952, Saudi Arabia

**Keywords:** Cytochrome *c*, Apoptosis, Apoptosome, Cancer, Warburg effect, Oxidative phosphorylation, And cancer

## Abstract

Cytochrome *c* is a vital electron carrier in the mitochondrial respiratory chain. When the outer membrane of mitochondria becomes permeable, cytochrome *c* is discharged into the cytoplasm, where it initiates the intrinsic apoptosis pathway. The complex interaction between cytochrome *c* and apoptosis protease-activating factor-1 (Apaf-1) leads to the formation of the apoptosome and activation of a cascade of caspases, highlighting the critical role of cytochrome *c* in controlling cell death mechanisms. Additionally, cytochrome *c* undergoes post-translational modifications, especially phosphorylation, which intricately regulate its roles in both respiration and apoptosis. These modifications add layers of complexity to how cytochrome *c* effectively controls cellular functions. cytochrome *c* becomes a lighthouse in the intricate web of cancer, its expression patterns providing hints about prognosis and paths toward treatment. Reduced levels of cytochrome *c* have been observed in cancer tissues, indicating a potential inhibition of apoptosis. For instance, in glioma tissues, cytochrome *c* levels were lower compared to healthy tissues, and this reduction became more pronounced in advanced stages of the disease. However, the role of cytochrome *c* in cancer becomes more intricate as it becomes intertwined with the metabolic reprogramming of cancer cells. This suggests that cytochrome *c* plays a crucial role in tumor progression and resistance to treatment. Viewing cytochrome *c* as a molecular mosaic reveals that it is not merely a protein, but also a central player in determining cellular fate. This realization opens up exciting avenues for potential advancements in cancer diagnosis and treatment strategies. Despite the advancements made, the narrative surrounding cytochrome *c* remains incomplete, urging further exploration into its complexities and the biological implications linked to cancer. cytochrome *c* stands as a beacon of hope and a promising target for therapy in the battle against cancer, particularly due to its significant involvement in tumor metabolism. It holds the potential for a future where innovative solutions can be developed to address the intricate challenges of cellular fate. In this review, we have endeavored to illuminate the multifaceted domain of cytochrome *c* drawing connections among apoptosis, metabolic reprogramming, and the Warburg effect in the context of cancer.

## Graphical abstract

**Figure:**
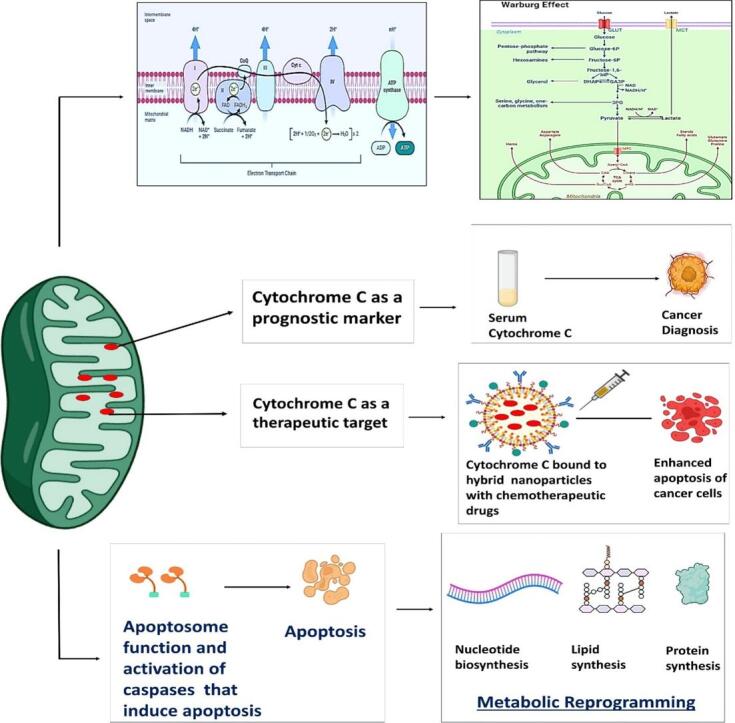

## 1 Introduction

Effective cancer therapy requires both very sensitive therapies to malignant cells and reliable prognostic indicators [1{Sofi, 2023 #463{Sofi, 2023 #463]}. It is essential to fully identify the metabolic alterations that malignant cells go through in order to achieve these two crucial goals (Yousuf, 2022). Such alterations may reveal chemicals with altered concentrations, adaptations, or intracellular locations. Monitoring the rate at which they return to pre-treatment levels is one technique to assess the efficacy of cancer treatments. Restoring a protein in cancer that has altered in level, location, and intracellular nature can therefore have a therapeutic effect (Sofi, 2022). Cytochrome *c* has emerged as a promising prognostic predictor and potential treatment agent as it is involved in many important apoptotic and metabolic pathways. According to recent studies, Cyt C is a compact globular protein housing an iron porphyrin cofactor known as heme c, which exhibits a sole polypeptide chain of 104-amino acid to which it is covalently attached, that directs critical choices between cellular survival and demise. Its primary role lies in participating within the Electron Transport Chain (ETC) situated on the inner membrane of mitochondria, thereby serving as a pivotal component crucial for cellular respiration maintenance (Chertkova, 2017). Cyt C is a versatile enzyme crucial for cellular energy production and apoptosis. It functions in the mitochondrial electron transport chain and is key to apoptosome formation. Recent research highlights its role as a cardiolipin peroxidase and reveals phosphorylation at four sites, indicating its regulation by cell signaling pathways. Understanding these pathways could be pivotal for developing therapies for neurodegenerative diseases, heart failure, and cancer (Hüttemann, 2011, Kalpage, 2019). Apoptosis, a meticulously regulated process of cellular self-destruction (Fogarty and Bergmann, 2015), serves as a cornerstone in physiological development, homeostasis, and immune defence, ensuring the removal of unwanted or damaged cells. Cyt C, which was first discovered to be an essential apoptotic mediator due to its connection to the intrinsic mitochondrial route, is at the centre of this complex apparatus (Wu and Bratton, 2013). When released from the mitochondria, Cyt C serves as an apoptotic signalling molecule. Further research has clarified the importance of Cyt C and shown how crucial it is for triggering downstream caspases and regulating apoptotic cascades (Bayir, 2006). Moreover, Cyt C's release from mitochondria serves as a biomarker for apoptosis (Manickam, 2017), offering insights into disease prognosis and treatment response across various cancers. It interacts with Apaf-1 to form an active apoptosome, activates caspase-9, and starts the cascade of caspases. (Wu, 2016). Reactive oxygen species is a significant inducer of intrinsic apoptosis (ROS). Cyt C is an electron carrier that contributes to the production and removal of reactive oxygen species (ROS) (Chen, 2003). Furthermore, it also catalyzes cardiolipin peroxidation (Hüttemann, 2011, Kalpage, 2019), a process that encourages Cyt C release from mitochondria, fostering apoptosis. Cyt C emerges as a significant target for the signaling of cancer cells, due to its dual roles in oxidative phosphorylation (OxPhos) and role in intrinsic apoptosis (Hüttemann, et al., 2014). Cyt C acts as a transporter of electrons in the ETC and it helps in transferring electrons from complex III to complex IV (COX) (Guo, 2018), where oxygen undergoes reduction to form water in the ETC. It stands as an essential component for generating ATP and ensuring cell survival (Garrido, 2006).

Cytochrome *c* can be released into the extracellular space by damaged or dying cells, potentially serving as a signaling molecule that alerts nearby cells to tissue damage. Elevated levels of serum cytochrome *c* have been observed in various conditions, such as inflammatory arthritis, myocardial infarction, and liver diseases. It may also indicate mitochondrial injury following events like heart failure resuscitation, snakebite envenomation, and chemotherapy. Cytochrome *c* released into the bloodstream can trigger immune responses in astrocytes through interaction with toll-like receptor 4 (TLR4), suggesting that targeting the cytochrome *c*-TLR4 pathway might help reduce inflammation resulting from cell death. Conversely, intravenous administration of cytochrome *c* in septic mice has been shown to restore cytochrome *c* oxidase activity in damaged myocardium, leading to a significant improvement in survival (Zhou et al., 2019).

Cancer cells have a distinct metabolic phenotype marked by dysregulated mitochondrial activity, enhanced glycolysis, and lactate generation (Oliveira, 2021). At the intersection of these metabolic pathways, cytochrome *c* plays a dual role in energy production by promoting oxidative phosphorylation and undergoing post-translational modifications that affect its activity and influence the metabolic changes seen in cancer cells (Peng, 2022). Unlike oxidative phosphorylation, aerobic glycolysis is favoured by the majority of cancer cells—a phenomenon called the “Warburg effect.” This change in metabolism is a part of the cancer cells' reprogramming technique to increase biomass synthesis in anabolically effective ways, which are necessary for strong cellular proliferation (Vander Heiden et al., 2009). The resistance to cell death and disruption of cellular energetics are prominent features of carcinoma (Hanahan and Weinberg, 2011). Cyt C is therefore situated at the centre of pathways governing both of these carcinogenic processes.

Currently, no research has looked into how changes to Cyt C's structure and function can affect tumour metabolism in cancer as a whole. Comprehending the molecular processes that dictate Cyt C's actions offers fresh perspectives on the biology of cancer and identifies potential targets for therapeutic intervention. Dysregulation of Cyt C expression and localization has been linked to carcinogenesis and the advancement of cancer. Research has shown that malignant tissues contain different levels of Cyt C, which may play a part in regulating apoptotic susceptibility and affecting tumour behaviour. Moreover, new data indicates that regulating Cyt C levels or promoting its release from mitochondria may have therapeutic potential, providing innovative methods for increasing cancer cells' sensitivity to apoptotic cues and strengthening treatment results. Even while the significance of Cyt C in cellular physiology and cancer biology has been clarified with great progress, there are still some unsolved problems. Undoubtedly, the future research endeavours will centre on unveiling the intricacies of Cyt C regulation, investigating its interplay with other cellular pathways, and utilising its therapeutic potential for the treatment of cancer. We can deepen our grasp of Cyt C biology and investigate potential avenues for novel approaches to cancer therapy by filling in these information gaps. We hope to shed light on Cyt C's significance for cancer pathogenesis and treatment approaches by thoroughly analysing its complex role in apoptosis and metabolism in this review. By means of a comprehensive review and critical evaluation, we outline the complex molecular pathways involving Cyt C and suggest avenues for future investigation in this emerging subject.

## 2 Cytochrome *c*: A key player in programmed cell death

**Fig. 1:**
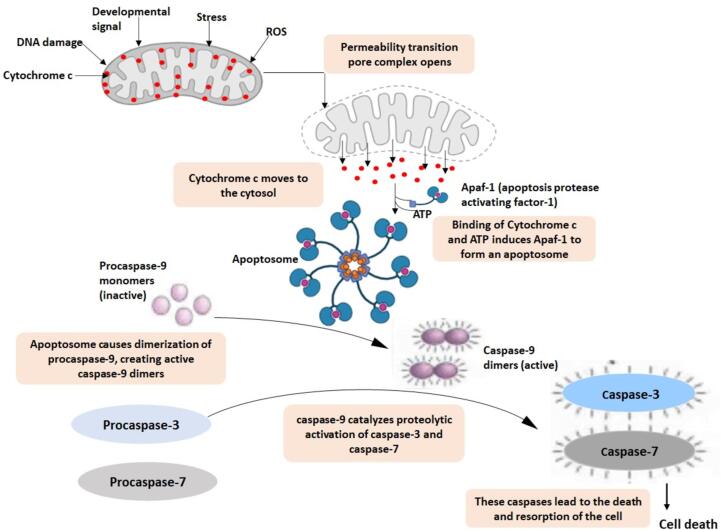
Role of cytochrome *C* in apoptosis. Numerous death signals gather at the mitochondria, releasing a variety of intermembrane space proteins. Numerous apoptotic stimuli, such as death domain receptors, chemotherapy, agents that damage DNA, withdrawal of growth factors, and radiation, cause mitochondria to become activated. This leads to the release of apoptotic proteins, such as endonuclease G, cytochrome *c*, AIF, Smac/DIABLO, and Omi/HtrA2. Cytochrome *C* binds to Apaf-1 to cause caspase activation. IAP inhibition of caspases can be counteracted by Omi/HtrA2 and Smac/DIABLO.

Apoptosis, or programmed cell death, is a vital process that eliminates unwanted cells from the organism (Mehraj, 2023). It is also important for immune system development, embryonic development, and regulating body temperature. Cyt C's role in PCD was first identified in experiments where dATP was added to cytosolic extracts to stimulate caspase activity (Liu, 1996). The process of separating the cytosol extracts revealed Cyt C to be the component of the caspase-inducing mechanism. When Cyt C was reintroduced, the potential of PCD was restored. Immune depletion of Cyt C from an extract removed its ability to induce apoptosis. The Xenopus egg-extract method was the first to demonstrate the mitochondrial connection, allowing for spontaneous caspase activation. Furthermore, it was shown that Cyt C is the main mediator of this effect (Kluck, 1997). After that, it was found that cytosolic microinjection of Cyt C into various mammalian cell types might trigger apoptosis (Li, 1997, Zhivotovsky, 1998). On the other hand,Cyt C-deficient cells showed less caspase-3 activation and tolerance to a variety of apoptotic triggers (Li, 2000). The main players in apoptosis are cysteine proteases called caspases, which break down cells in a coordinated cascade **(**Fig. 1**).** The cascade of caspases consists of “initiator” caspases and “executioner” caspases, which are each triggered differently by different apoptotic triggers (Mehta et al., 2007).

Mitochondrial Cyt C has multiple roles in controlling apoptosis and cellular energy metabolism (Zou, 1999). It is commonly known that mitochondria play a critical role in energy metabolism, ion balance, and redox regulation. Damage to these organelles is always linked to cellular death. (Reed, 1997). Disruption of cellular death pathways such as autophagy and apoptosis is a major element in carcinomas' resistance to both radiotherapies and chemotherapy (Kornienko, 2013). A decrease in the levels of certain pro-apoptotic proteins and an increase in the levels of proteins that prevent apoptosis are the outcomes of this disruption. More recently, the majority of apoptosis research has focused on altering the respiratory chain of mitochondria rather than altering the nuclear structure (Lemarie and Grimm, 2011). Apoptosis reliant on caspases transpires through two primary routes: the intrinsic pathway and the extrinsic pathway (Riedl and Salvesen, 2007) **(**Fig. 1**)**. The intrinsic mitochondrial pathway is set in motion by certain triggers causing the mitochondrial outer membrane permeabilization (MOMP) and the liberation of proteins from the intermembrane space of mitochondria. Within this, numerous proteins contribute to triggering the activation of caspases, with Cyt C being one such protein. Within the inner mitochondrial membrane (IMM), utilizing its heme moiety, Cyt C actively participates in the electron transfer pathway, shuttling electrons between Complex III and Complex IV within the ETC. Upon sensing an apoptotic trigger, like DNA harm, metabolic strain, or the presence of unfolded proteins, the intrinsic pathway of apoptosis is activated, causingCyt C to be discharged into the cytoplasm and therefore interacts with APAF1(Fig. 1) (Liu, 1996, Kluck, 1997, Kluck, 1997, Yang, 1997). The initiation of the extrinsic apoptotic pathway begins with the binding of multiple transmembrane death receptors (e.g., FAS) to an extracellular ligand (e.g., FASL), leading to the creation of the death-inducing signaling complex (DISC). Following this, the DISC serves as a trigger for initiator caspases, which set off an enzymatic cascade ultimately leading to PCD.

CCyt C plays a crucial role in apoptosis triggered by mitochondria and serves as a significant element in energy metabolism, forming an essential part of the respiratory chain. Liu et al. (Liu, 1996) first proposed the role of Cyt C in apoptosis. After being liberated into the cytoplasm, Cyt C interacts with its adapter molecule Apaf-1 to trigger the activation of pro-caspase-9 in ATP’s presence. Activated cas-9 then activates cas-3, resulting in the molecular traits of apoptosis via the intrinsic mitochondrial route **(**Fig. 1**)** (Anguita and Villalobo, 2018). An essential initial stage in the advancement of apoptosis entails the liberation of Cyt C from the mitochondria into the cytoplasm. Serving as a constituent of the mitochondrial ETC, Cyt C assumes a crucial function in electron transfer between complex III and complex IV (Cai et al., 1998). Within 60 min of apoptosis induced by compromised mitochondria permeabilization, Cyt C, an indicator of mitochondrial integrity, is released into both the extracellular space and the bloodstream (Renz, 2001). As a result, Cyt CCyt C is recognized as a vital mediator and marker in intrinsic apoptosis **(**Fig. 1**)**.

The existing body of literature has demonstrated that serum Cyt C anticipates the prognosis across various cancer treatments, encompassing conditions such as leukemia, lung cancer, and breast cancer (Wen, 2014, Barczyk, 2005, Osaka, 2009) (Santidrian, 2013). This suggests that Cyt C might contribute to both the initiation as well as the progression of carcinogenesis. Avoiding apoptosis stands out as a fundamental characteristic of cancer. The inception, progression, and spread of tumors result from oncogenic occurrences, including the down-regulation of tumor suppressor genes or the up-regulation of oncogenes, which disrupt the process of apoptosis (Vladimirov, 2017). The mitochondrial signaling pathway is implicated in the liberation of Cyt C and proteins inducing mitochondrial apoptosis through the Bcl-2/Bax axis, thereby initiating subsequent phases of apoptotic mechanisms (Kao, 2017, Irizarry Rovira, 2018, Trotta and Chipuk, 2017) Consequently, Cyt C may play a part in the initiation and advancement of cancer.

## 3 Cytochrome *c* Modulates histone Chaperone function by inhibiting nucleosome assembly

Nur-E-Kamal et al. were the first to observe that Cytochrome *C* (Cyt C) moves into the nucleus following DNA damage, even in the absence of caspase activation. They proposed that Cyt C might be involved in chromatin remodeling, but its specific nuclear role was not fully understood. Their findings confirm that Cyt C indeed translocates to the nucleus in response to double-strand breaks (DSBs) before caspase activation (Nur-E-Kamal, 2004). Crucially, they have discovered that nuclear Cyt C interferes with the binding of several chromatin-associated proteins—namely, SET/template-activating factor Iβ (SET/TAF-Iβ), acidic leucine-rich nuclear phosphoprotein 32 family member B (ANP32B), nucleolin, and NAP1-related protein (NRP1)—to core histones, thereby inhibiting their nucleosome assembly function. Both Cyt Cand histones have highly positive isoelectric points, allowing them to interact with the low-complexity acidic regions (LCARs) of these chaperones. Low-complexity regions (LCRs) in proteins are characterized by minimal sequence diversity and are often rich in acidic residues. Specifically, the term LCAR refers to low-complexity acidic regions, which are involved in binding interactions with proteins. Recent studies have defined 'acidic domains' as sequences with at least 20 acidic residues within 50 consecutive amino acids and suggested that these domains are prevalent in histone-binding proteins due to their flexible and minimal structural requirements for binding. Interactions between Cytochrome *C* (Cyt C) and LCAR-containing chromatin-associated proteins (Chs) are transient and electrostatic. Cyt C binds to various proteins, including human SET/TAF-Iβ and plant NRP1, via the same surface patch around its haem cleft. This binding mechanism is conserved and involves interactions with proteins in both the electron transport chain and apoptosis pathways. Cyt C plays a regulatory role in chromatin remodeling during DNA damage response (DDR) by sequestering LCAR-containing Chs, such as SET/TAF-Iβ and NRP1, thereby potentially modulating DNA repair processes. These proteins, despite their structural and functional similarities, are inhibited by Cyt C, which may help separate transcription from DNA repair. Additionally, SET/TAF-Iβ is involved in regulating DNA repair and transcription, and its inhibition by Cyt C might coordinate these processes effectively (González-Arzola, 2022, Rivero-Rodríguez, 2021).

## 4 Cellular translocation of cytochrome *c*: Unravelling the molecular pathways

**Fig. 2:**
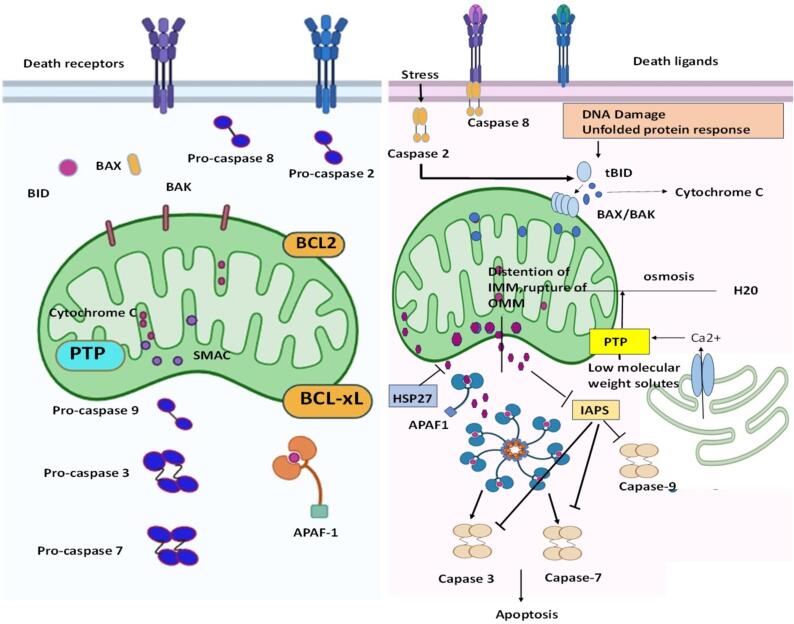
The release of cytochrome *c* from mitochondria and its subsequent downstream processes. When cytochrome *c* is released from mitochondria, it triggers a cascade of events leading to apoptosis, or programmed cell death. This release is tightly regulated and can be influenced by various factors including cellular stress, mitochondrial dynamics, and apoptotic signaling pathways.

During mobilisation,Cyt C separates from the IMM and separates from the membrane phospholipid cardiolipin by oxidising cardiolipin (Ott et al., 2007, Gogvadze et al., 2006). Within the IMM, Caspase-2 shatters the link between cardiolipin and cytochrome *C* (Gogvadze et al., 2006), possibly amplifying the mobilization of cytochrome. The increased creation of ROS can intensify the movement ofCyt C (Garrido, 2006, Kroemer et al., 2007). Cyt C's mobilisation may also include it leaving tightly packed cristae junctions **(**Fig. 2**)**. To explain the swift and wide-ranging discharge ofCyt C during apoptosis (Goldstein, 2000), it has been proposed that cristae undergo remodeling, thereby redistributing cytochrome *c* within the mitochondria before its translocation through the OMM (Scorrano, 2002). The mobilised protein is transferred from the OMM to the cytosol in the next step of Cyt C release **(**Fig. 2**)**. BCL2 family death agonists cause MOMP, which controls mitochondrial integrity and, in turn, regulates Cyt C release and intrinsic mitochondrial apoptosis (Youle and Strasser, 2008). The BCL2 family's effector proteins, like BAX and BAK, are necessary and sufficient for MOMP, and the process does not occur in their absence (Wei, 2001, Chandra, 2005). Heat shock protein-27 (HSP27), potentially by shielding the filamentous actin network, prevents BID from moving intracellularly, which prevents Cyt C from being released (Paul, 2002) **(**Fig. 2**)**. HSP27 binds to Cyt C within the cytoplasm, blocking any further effects. Additional heat-shock proteins, like HSP70 (Garrido, 2006) and HSP72 (Steel, 2004) have similarly been demonstrated to affect the discharge of Cyt C. When calcium ions (Ca2+) are released from the extracellular space (ER), the permeability transition pore is activated, leading to a wider release of cysteine (Wei, 2001, Mootha, 2001).

In numerous cell varieties, the liberation of Cyt C during PCD occurs swiftly, fully, and irreversibly (Goldstein, 2000). The liberation of Cyt C during MOMP is believed to be biphasic. An amplification loop and the modification of mitochondrial cristae precede the release of a “loosely tethered” reservoir, which in turn triggers the release of a more firmly tethered reservoir (Garrido, 2006, Scorrano, 2002). The loosely tethered'soluble' Cyt C pool is responsible for both electron transport and ROS regulation. In other terms, it's important for mitochondrial OXPhos and ATP synthesis. Interestingly, there appears to be no discernible disruption of these processes until the release of the second wave of Cyt C, suggesting that metabolic compensation may occur during incomplete MOMP. This leads to respiratory collapse by escalating the generation of ROS (Garrido, 2006). The results show that caspase-3 begins cleavage of the p75 subunit (NDUFS1) within Complex I subsequent to Cyt C's release. This results in a further reduction in the integrity and potential of the mitochondrial membrane, increased ROS production, damage to the plasma membrane, and a catastrophic drop in ATP levels (Ricci, 2004). Thus, the degree ofCyt C release controls apoptotic involvement in addition to oxidative metabolism and ATP synthesis. This demonstrates how important it is for MOMP levels to regulate the balance between the apoptotic and metabolic reactions to cellular stress.

## 5 Cytochrome *c* dynamics and Warburg's Principle: A metabolic Perspective

It is becoming more widely recognised that the metabolic reprogramming that occurs in tumour cells is a major cause of disease and regulates many aspects of malignant growth (Buchakjian and Kornbluth, 2010). Mutations in genes related to mitochondrial metabolism set off the stress response pathway, which in turn helps to initiate carcinogenesis (Wallace, 2012). Changes in the redox state of the cell and increased production of ROS by the mitochondria can affect the way transcription factors that encourage the growth of cancer cells work (Ishikawa, 2008). Anomalies in mitochondrial function and irregular molecular expression regulating metabolism of energy have been documented in various cancer types, including gliomas (Ordys, 2010), prostate (Grupp, 2013), and breast cancer (Santidrian, 2013). The adjustment of energy metabolism in tumor cells has been proposed as a pivotal factor supporting the proliferation of cancer cells at metastatic locations (Santidrian, 2013). The operational function of molecules within the mitochondrial ETCplays a vital role in this mechanism. The ETC serves as the location for oxidative phosphorylation, supplying the energy needed for ATP synthase. Additionally, the ETC is a significant point where electrons may prematurely leak into oxygen, thereby generating superoxide and potentially leading to heightened oxidative stress. The concluding complex of ETC, COX, facilitates the transfer of electrons from Cyt C to the molecular oxygen and is considered the crucial regulatory site for OXPhos. Elevated consumption of glucose for energy, attributed to modified mitochondrial function, is presently recognized as a characteristic feature of numerous rapidly proliferating tumors (Ward and Thompson, 2012, Koppenol et al., 2011). Nevertheless, diverse types of cancer cells experience distinct bioenergetic modifications: some lean towards a heightened glycolytic state, while others prefer the channelling of fuels through OXPhos (Kadenbach, 2000). Therefore, examining mitochondrial characteristics unique to a given tissue is essential to understanding the metabolic changes occurring in cancer cells.

**Fig. 3:**
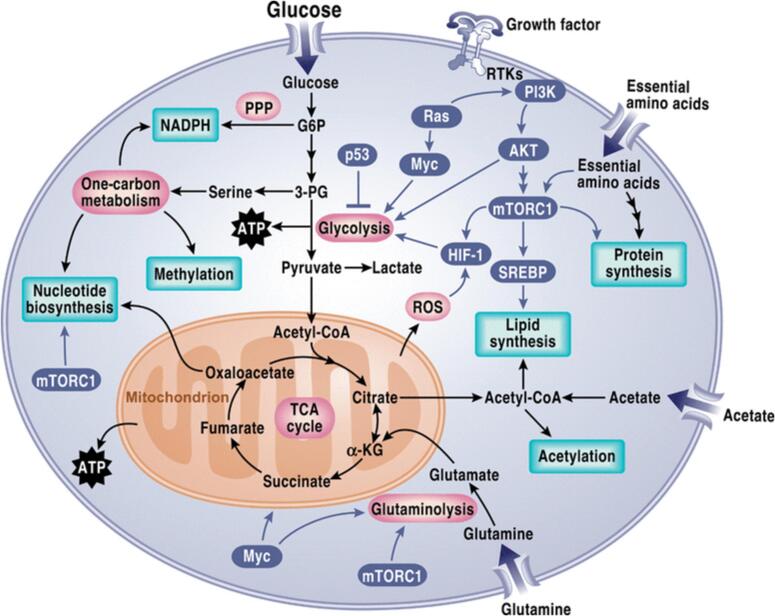
The diagram illustrates the metabolic reprogramming observed in cancer cells, highlighting key alterations such as increased glutamine metabolism, enhanced glycolysis with lactate fermentation, perturbations in the tricarboxylic acid (TCA) cycle, utilization of intermediates from the pentose phosphate pathway, and the synthesis of lipids, amino acids, and nucleotides utilizing TCA cycle intermediates.

Metabolism of glucose stands out as the most widely recognized varied metabolic route in cancer cells (Kalyanaraman, 2017). Subsequently, numerous other pathways have been identified as being modified in cancer, including oxidation of fatty acids, acquisition of lipids, glutamine metabolism, one-carbon metabolism, metabolism of branched-chain amino acids, and the Krebs cycle (Läsche et al., 2020) **(**Fig. 3**)**. The rewiring of these pathways entails complex mechanisms and the coordination of diverse signaling molecules, involving molecules once thought to be insignificant, such as non-coding RNAs. Hypoxic conditions within the tumor microenvironment (TME) induce metabolic shifts, such as the Warburg phenomenon, in cancerous cells. Changes in the TME caused by hypoxia prompt transcriptional responses through HIF activation under stress conditions. This leads to alterations in metabolic pathways within cancer cells (Kaelin and Ratcliffe, 2008). On the cell membrane, PI3K phosphorylates and triggers the activation of AKT **(**Fig. 3**)**, a significant signaling molecule that instigates the Warburg phenomenon in cancerous cells (King et al., 2015). Activation of mTOR by AKT enhances lipid synthesis and facilitates glucose transportation to cancer cells, thereby enhancing glycolysis. The PI3K/AKT pathway stimulates the metabolism of cancer cells by promoting glucose uptake, glycolysis, and lactic acid generation. This pathway plays a crucial role in the metabolic reprogramming of cancer cells (Polivka and Janku, 2014). Glutamine serves as an additional carbon source in biosynthetic pathways, supporting fatty acid synthesis. α-Ketoglutarate derived from glutamine **(**Fig. 3**)** fuels citrate production via the forward flux in the TCA cycle and contributes to pyruvate generation through malic enzyme-dependent pathways (DeBerardinis, 2007). Furthermore, because these mechanisms are complex, metabolic reprogramming pathways in cancer frequently occur to varying degrees and in different situations across carcinomas, providing cancer cells with flexibility not observed in normal cells (Schiliro and Firestein, 2021). Regular cells often undergo PCD as a normal part of the cell cycle, while malignant cells avoid apoptosis in order to promote their growth, maturation, and expansion—especially in low-oxygen environments. Metabolic reprogramming is closely linked to several mechanisms used by cancer cells to avoid apoptosis. One prominent characteristic of cancer is the metabolic reprogramming that is marked by increased glycolysis, especially in glucose metabolism (Ghanavat, 2021). Through a number of channels, the avoidance of apoptosis and glucose metabolism are closely related. A number of signalling molecules that are involved in increasing glycolysis are also involved in inhibiting apoptosis. Among the chemicals involved in the metabolism of glucose is Cyt C, which also has an effect on apoptosis suppression. Cyt C starts the mitochondrial apoptotic pathway, however because of glucose metabolism, it changes. Because of the increased flow in the pentose phosphate pathway (PPP) in cancer cells, there is a greater production of NADPH, which prevents Cyt C from activating by keeping it in its decreased, inactive state (Vaughn and Deshmukh, 2008). Glycolysis in cancer cells is additionally controlled by Akt, which impedes PCD by restraining two BCL-2 pro-apoptotic family proteins, namely glycogen synthase kinase 3 (GSK-3), p53 upregulated modulator of apoptosis (PUMA). Additional substances involved in glucose metabolism and apoptosis inhibition include BCL2-associated agonist of cell death and TIGAR, the tp53-induced glycolysis and apoptosis regulator (BAD) (Yu, 2004).

Two classic traits of malignant cells are resistance to cellular death and a metabolic switch from OxPhos to glycolysis, known as the Warburg effect. Cyt C is located at the intersection of these two pathways. According to the Warburg Effect, aerobic glycolysis—the process by which glucose is changed into lactate in the presence of oxygen—is the primary means by which malignant cells produce ATP (San-Millán and Brooks, 2017). This is in contrast to normal cells, which primarily depend on OXPhos for the production of ATP (Warburg, 1925). Since Warburg's concept was put forth, our knowledge of the function of aerobic glycolysis in malignant cells has advanced significantly. In particular, an elevated ratio between oxygen consumption and glycolysis has been noted; this is a result of modifications in oxidative metabolism, the downregulation of tumor-suppressive genes, and the upregulation of genes that support cancer (Pascale, 2020). One of the most important aspects of malignant cells is their metabolism of glucose, which supplies the building blocks for many vital metabolic pathways, including the creation of lipids, amino acids, and nucleotides (Vander Heiden et al., 2009).

**Fig. 4:**
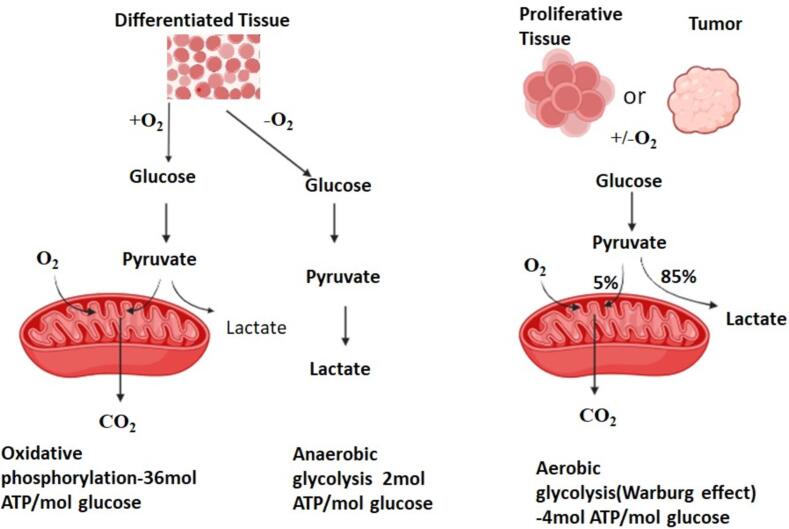
In the presence of oxygen, tumors and other highly proliferative cells preferentially convert most of their glucose into lactate through a process known as the Warburg Effect. Despite yielding approximately 4 molecules of ATP per molecule of glucose, this pathway provides cancer cells with a significant growth advantage over oxidative phosphorylation (OXPhos) due to its faster chemical reaction. In normally differentiated tissues, glucose undergoes conversion to pyruvate when oxygen is available. Pyruvate then enters oxidative phosphorylation (OXPhos), generating approximately 36 molecules of ATP per molecule of glucose. Alternatively, when glucose oxidation does not occur, lactate is produced, yielding about 2 molecules of ATP per molecule of glucose.

Consequently, one of the primary inquiries regarding the Metabolism of glucose in cancer cells revolves around the question of why cancerous cells would favour a mechanism of energy generation that has comparatively low efficiency since OXPhos produces 36 ATPs per glucose molecule, glycolysis only yields 2 ATPs **(**Fig. 4**)**. The reason for this is that cytosolic ATP synthesis occurs at a rate that is roughly 100 times (from 20 to 300 times) faster than that of mitochondria, which are known for their “high-speed ATP generation, but low yield” (Vaupel et al., 2019). Thus, aerobic glycolysis can increase quickly when cancer cells need a large quantity of ATP, yet OxPhos stays relatively constant. This is due to the Warburg effect, which significantly speeds up ATP synthesis (Läsche et al., 2020). For cancer cells to achieve the uncontrolled growth characteristic of their nature, rapid energy generation is imperative, and aerobic glycolysis effectively fulfils this requirement. Moreover, the substantial production of lactate contributes to an acidic microenvironment, fostering the growth of cells with phenotypes resistant to acidity. This confers a significant growth advantage, intensifying the invasive and rapidly dividing tendencies of cancerous cells as neighbouring cells deteriorate (Ghanavat, 2021). Extracellular acidosis, with a pH level below 6.8, is a harmful characteristic that occurs as a result of both aerobic and anaerobic glycolysis. The primary function of this is to facilitate the advancement of cancerous growth and resistance to traditional treatments (Riemann, xxxx). Cancer cells require significant quantities of glucose to maintain energy homeostasis.

Numerous important functional and molecular processes, including a marked increase in the rate of glycolytic flows, the generation of enough ATP in a certain amount of time to maintain the energy equilibrium, and the diversion of intermediates from glycolytic processes to support the synthesis of lipids, hexosamines, non-essential amino acids, and nucleotides, are characteristics of the Warburg effect (Burns and Manda, 2017). It also entails an increased amount and accumulation of lactate, as well as the inhibition of pyruvate from entering mitochondria. In addition to encouraging tumour growth and inhibiting anti-tumor immunity, lactate may also provide cancer cells with energy in oxygen-rich settings, which increases the likelihood of malignancy and makes conventional treatments more difficult to administer (Wang, 2021). A metabolic program of cancer cells drives prolonged growth and accelerates aggressive progression; this is reflected in the Warburg effect (Vaupel et al., 2019). The Warburg effect facilitates the provision of reducing equivalents, which in turn promotes the development of cancer cells. For every glucose molecule, the oxidative branch of the PPP produces two NADPH molecules. This keeps glutathione's antioxidative potential intact, which strengthens the cancer cells' resistance to radiation. Furthermore, NADPH might have an antioxidant role in the mitochondria (Kirsch and De Groot, 2001). Moreover, active PPP can lessen ROS production, which at lower concentrations can increase cancer cell survival. Moreover, NADPH is needed for reductive biosynthesis, which produces fatty acids, which are necessary for the synthesis of membrane lipids (Bazylianska, 2021).

The transformation of glucose to lactate in the presence of oxygen and functional mitochondria, known as the Warburg effect (Gatenby and Gillies, 2004), is undoubtedly more than just an adaptation to hypoxia. On the contrary, it is an urgent component of cancer's phenotype and a key aspect of cancer cells' “selfish” metabolic reprogramming, a trait sometimes referred to as a “hallmark of cancer.” (Hanahan and Weinberg, 2011). Changes in glucose metabolism in cancer therefore promotes the growth of the disease by stimulating growth-promoting pathways as well as by preventing cell death and strengthening survival.

## 6 Cytochrome *C* expression patterns in tumor Heterogeneity: Clinical and therapeutic perspectives

**Fig. 5:**
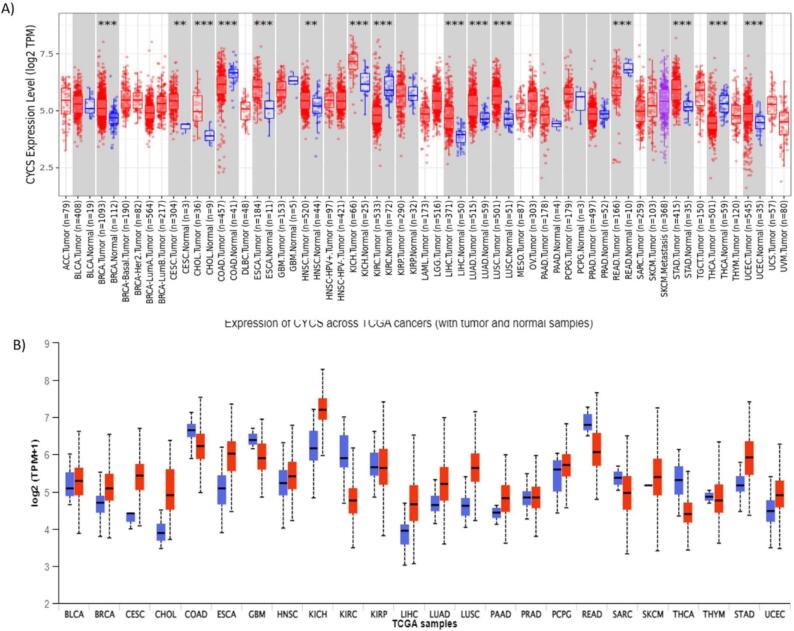
The bioinformatic analysis of Cyt C using online portals. (A) Timer Analysis showing the expression pattern of Cytochrome *c* in various cancers which reveals that Cytochrome *c* expression is highly upregulated in several cancers including breast cancer. (B) Gepia-2 Analysis further validates that Cytochrome *c* is dysregulated in several malignancies including Breast Cancer.

The amount of Cyt C that is released can affect what happens next. Studies have demonstrated that a living rat brain cell can tolerate laser micro-irradiation damage to about 15 % of its mitochondria. There wasn't enough Cyt C produced by these mitochondria to activate caspase-9. (Khodjakov, 2004). This suggests that caspase-9 is inactive below a certain critical concentration of Cyt C. Depending on the type of cell, this threshold may vary greatly. This could be because different regulators, such as inhibitors of apoptosis proteins (IAPs), affect how much caspase activity is modulated. Rana et al. (Rana, 2022) have reported that the levels of Cyt C expression are diminished in glioma tissues compared to those in normal tissues. The fact that the expression level decreases as glioma grades rise only heightens the intrigue. As a result, our finding suggests that Cyt C may be a target for prognostic biomarkers in gliomas. Furthermore, it was observed that overexpression of Cyt C prevented the growth of tumours in a mouse model of clear cell renal cell carcinoma (Liu, 2019). On the other hand, a clear cell renal cell carcinoma cell line's proliferation was boosted by Cyt C suppression, most likely as a result of stopping programmed cell death. In these cells, Cyt C overexpression accelerated the rate of apoptosis (Liu, 2019). In addition, numerous commonly used chemotherapeutic medications trigger apoptosis by facilitating the release of Cyt C into the cytoplasm (Delinois, 2021). Thus, the rise in cytoplasmic Cyt C levels is directly related to the ability to fight the disease. The examination of Cyt C expression levels in various cancers is yet largely unexplored, offering intriguing opportunities for future research. This study project has the potential to establish Cyt C as a useful and reliable prognostic marker in cancer biology, which would be a major advancement in the field. Fig. 5 shows the expression pattern of Cyt C in various types of cancers.

## 7 Regulatory roles of cytochrome *c*: Post-Translational modification

Cytochrome *c* (Cc) is an essential component of cellular metabolism and apoptosis, and post-translational changes play a crucial role in controlling its actions (PTMs). These changes have the potential to significantly change Cc's stability, activity, and interactions with other proteins, which could have an impact on important biological pathways. Phosphorylation, which can take place at particular serine or threonine residues, is one important PTM (Kalpage, 2019). By improving cytochrome *c*'s binding affinity to apoptotic factors such as Apaf-1, this alteration can facilitate apoptosome activation and promote programmed cell death. This reaction is essential for removing strained or injured cells and preserving tissue equilibrium (Lee, 2006, Mahapatra, 2017).

Another important alteration that affects the function of cytochrome *c* is acetylation. The stability of cytochrome *c* can be improved by adding acetyl groups, especially to lysine residues, which will change where it is located in the mitochondria. Additionally, acetylation can affect how it interacts with proteins in the electron transport chain, which can change metabolic pathways and energy generation (Morse, 2024).

## 8 Furthermore, cytochrome *c*'s oxidative alterations can change the heme group's redox state, which can impact the enzyme's ability to transfer electrons. This alteration is particularly pertinent in the setting of oxidative stress, since reactive oxygen species (ROS) can cause modifications that either improve or impair the function of cytochrome *c*. When cytochrome *c* is oxidised, it can lose its capacity to engage in electron transport, interrupting ATP generation and potentially leading to cell death. The effects of these PTMs are extremely specific to the residues changed. For example, phosphorylation at one site may boost apoptotic signalling, while another site can hinder interactions with pro-survival proteins (Bazylianska, 2021). Because of its selectivity, cytochrome *c* is able to combine signals from several routes and play a crucial role in controlling apoptosis and cellular stress responses in addition to being a link in the electron transport chain. In summary, the regulatory responsibilities of cytochrome *c* are dramatically altered by post-translational changes, which allow for a nuanced response to cellular circumstances. By adjusting Cc’s activity through phosphorylation, acetylation, and oxidative alterations, cells can finely tune their metabolic responses and apoptotic signalling, underscoring the relevance of PTMs in maintaining cellular integrity and function. Exploring the future of cytochrome *c* targeting in Cancer: Prognostic and therapeutic Innovations

Cytochrome *C* (Cyt C) plays a critical role in apoptosis, and several aspects of its function are targeted in cancer treatment. Key sites include the heme group, which is essential for its electron transfer role, and the regulatory proteins controlling its release from mitochondria, which can be targeted to induce apoptosis. Hotspots of interest are the interaction sites where Cyt C binds to Apaf-1 to form the apoptosome and activate caspase-9, crucial for initiating apoptosis. Additionally, while Cyt C itself has limited phosphorylation, targeting the phosphorylation of associated proteins that regulate its release and apoptosome formation can influence its apoptotic function and provide potential therapeutic strategies in cancer treatment. Cytoplasmic Cyt C is able to get out of the cell. According to reports, Cyt C was constantly released from epithelial cells and entered the malignant duct lumen in breast cancer samples. Because Cyt C can elude cells, it can enter the bloodstream. (Abramczyk et al., 2022). As such, the amount present in the cytoplasm can be determined by measuring its concentration in the serum. It has been discovered that lower blood levels of Cyt C are caused by cancer. The serum levels of Cyt c were nearly three times lower in patients with non-small cell lung cancer than in healthy persons (Javid, 2015). The levels of Cyt C were reduced in the serum of patients with clear cell renal cell carcinoma (Liu, 2019). These data suggest that the lack of apoptosis induction in cancer may be caused by insufficient levels of Cyt C in the cytoplasm. Serum Cyt C levels, however, can range between cancer patients. People with clear cell renal cell carcinoma who had higher levels of Cyt C in their blood had better survival rates than those with lower levels of the protein, even though there was no discernible correlation between the protein's levels and the stage of the disease (Liu, 2019).

**Fig. 6:**
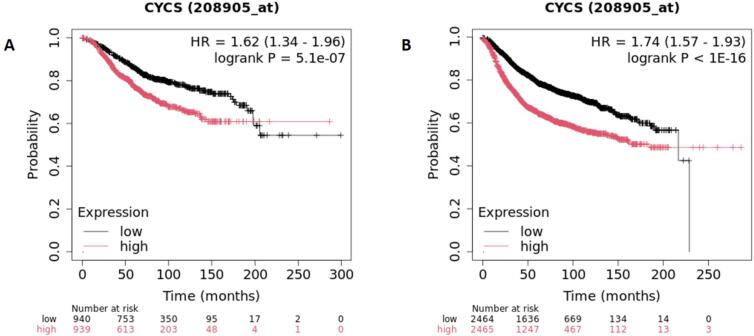
Bioinformatic survival analysis using KM plotter database. A) Overall survival of breast cancer patients. The survival probability increases with decrease in the level of cytochrome *c* and vice versa. B) The Relapse Free Survival of breast cancer patients. High mRNA expression of Cytochrome *c* is associated with worse RFS, with a hazard ratio reaching up to 1.74 in breast cancer.

It was discovered that individuals with various types of cancer who had elevated serum concentrations of Cyt C had a better chance of survival **(**Fig. 6**)**. Elevated serum Cyt C levels have been linked to an increased risk of neoplasm content; nevertheless, their rise after cancer therapy has been linked to a better prognosis (Barczyk, 2005). Elevated blood levels of Cyt C can also indicate heightened levels of apoptosis produced by chemotherapy (Barczyk, 2005). In patients with non-small cell lung carcinoma (Javid, 2015), the level of serum Cyt C raised a minimum of 13 times following the initial cycle of treatment. Chemotherapy has the potential to increase the levels of serum Cyt C in individuals with malignancies of the blood, such as acute myeloid leukemia and non-Hodgkin lymphoma (Renz, 2001). Patients with non-small cell lung carcinoma who had elevated amounts of Cyt C before chemotherapy saw a greater spike in Cyt C levels following the treatment (Javid, 2015). These findings imply that chemotherapy caused the patients' serum Cyt C levels to rise and their tumour PCD levels to increase. Chemotherapeutic drugs induce permeability in the mitochondrial outer membrane, releasing Cyt C into the cytoplasm and extracellular medium. As a result, the blood's concentration of Cyt C rose. It has been proposed that serum Cyt C levels are more sensitive in adult T-cell leukaemia patients (Renz, 2001) The prompt reaction of Cyt C levels to chemotherapy that induces PCD renders this protein an ideal indicator for evaluating the effectiveness of cancer treatments (Singh et al., 2019). Research has demonstrated that the combination of widely used anti-cancer drugs (vinblastine, vincristine, doxorubicin, paclitaxel, oxaliplatin, etc.) with hybrid nanoparticles bearing Cyt C considerably increases apoptosis in cell lines, ultimately leading to cellular death. As such, this combination strategy is promising for future treatment regimens (Al-Shakarchi, 2018).

### 8.1 Innovative biosensor technologies for serum cytochrome *C* Quantification

A range of advanced biosensors has been developed for quantifying cytochrome *c* (Cyt C) in human serum, with ELISA being one of the most commonly used methods. However, novel biosensors that do not rely on antibodies have been introduced, employing various detection techniques. Some of these sensors detect Cyt C based on its electrical properties. For example, certain biosensors use Cyt C oxidase linked to electrodes, where electron transfer from Cyt C to the oxidase alters electrical currents (Pessoa, 2022). Other electrochemical biosensors involve Cyt C binding to specific nanocavities, which affects the electrical current between an electrolyte solution and a gold electrode. Additionally, biosensors utilizing aptamers for Cyt C detection employ methods such as differential pulse voltammetry, surface-enhanced Raman scattering (SERS), and fluorescence (Tatarko, 2023). In differential pulse voltammetry, an aptamer immobilized on a glassy carbon electrode with carbon nanofibers allows Cyt C binding to transfer electrons to the electrode, generating an electrical signal. The SERS biosensor involves an aptamer hybridized with a fluorescently labeled oligonucleotide on a gold nanoparticle. Binding of Cyt C to the aptamer causes it to dissociate from the oligonucleotide, reducing the SERS signal due to increased distance from the gold nanoparticle. In the fluorescence-based biosensor, the aptamer on a graphitic carbon nitride nanosheet initially quenches fluorescence, which increases upon Cyt C binding as the aptamer dissociates from the nanosheet. Other biosensors use quantum dots, where Cyt C binding quenches the fluorescence. One such system also detects trypsin activity, as Cyt C proteolysis by trypsin restores fluorescence. Another quantum dot-based biosensor shows a more significant fluorescence decrease with Cyt C binding at higher temperatures. Additionally, there is a biosensor where trypsin is immobilized in porous nanostructures; Cyt C binding leads to its proteolysis by trypsin, producing a reaction product that diminishes the reflected light intensity. These biosensors exploit unique characteristics of Cyt C, including its binding to aptamers or the electrical currents generated from its heme group (Pessoa, 2022).

## 9 Cytochrome *c* impact on apoptosis and cellular metabolism in Cancer: Insights and implications

Extensive research on Cyt C has yielded significant insights into both the respiration of mitochondria (Pérez-Mejías, 2019) and Apoptosis (Kluck, 1997, Yang, 1997) Upon exposure to proapoptotic signals, the outer membrane of the mitochondria becomes permeable, leading to the release of Cyt C from the mitochondria into the cytosol. Inside the cytosol, Cyt C binds to Apaf-1 (Zou, 1997), initiating a chain of biochemical reactions that trigger the caspase cascade, a group of enzymes that promote apoptosis by tearing down cellular components (Zou, 1997, Slee et al., 2001). Apoptosis is a highly significant mechanism in the context of cancer. Firstly, cancer cells typically exhibit inhibition (Mohamed, 2017). Furthermore, the initiation of cancer cell apoptosis holds remarkable therapeutic promise (Pfeffer and Singh, 2018). MOMP is regarded as an irreversible event that initiates the intrinsic pathway of PCD (Singh et al., 2019). An immediate result of this is the release of Cyt C from the gap between the mitochondrial membranes into the cytoplasm. Reduced concentrations of Cyt C have been identified in cancerous tissues, indicating the suppression of apoptosis. Transformations in cancer-related metabolic pathways are dynamic, display adaptability, and frequently hinge on the tumor type and its microenvironment, this results in metabolic flexibility. Comprehending the intricate character of the changed flow through the various pathways in cancer cells can aid in the creation of novel therapeutic approaches (Schiliro and Firestein, 2021). The examination of Cyt C's function in cellular apoptosis holds significance not only in its own right but also in clinical diagnostics, where its presence in the cytoplasm serves as a valuable biomarker (Fiorucci, 2019). In instances of cellular injury, Cyt C released into extracellular regions may mimic other intrinsic danger-associated molecular pattern molecules (DAMPs), assuming a similar function when located in unintended compartments (Marenzi, 2016). Hence, the identification of Cyt C in extracellular spaces emerges as a noteworthy indicator for discerning severe mitochondrial impairment leading to cell demise.

Moreover, metabolic reprogramming in cancer, notably the Warburg effect, drives aggressive progression by shifting energy metabolism towards aerobic glycolysis, favouring rapid ATP production and lactate generation. This altered metabolism not only supports cancer cell growth but also confers resistance to apoptosis, fostering tumor survival. Dysregulated glucose metabolism, accompanied by mutations in genes associated with mitochondrial function, promotes tumorigenesis and sustains malignant advancement. For instance, lysine 53 acetylation of cytochrome *c* in prostate cancer cells alters its function, supporting the Warburg effect and helping the cells evade apoptosis. This modification contributes to cancer progression and resistance to therapy, highlighting potential targets for new treatments (Bazylianska, 2021). Additionally, oxidative stress and modifications in redox balance further contribute to tumor proliferation. Despite the diverse bioenergetic adaptations observed in different cancer types, targeting key metabolic pathways, such as glycolysis and the PPP, emerges as a promising strategy for cancer therapy. Moreover, the interplay between metabolism and apoptosis underscores the intricate mechanisms driving cancer progression, highlighting the multifaceted nature of the disease. Overall, understanding the intricate metabolic alterations in cancer cells offers valuable insights into therapeutic interventions aimed at disrupting tumor progression with Cyt C emerging as a possible target for novel treatment strategies. By targeting Cyt C and restoring its apoptotic function, it may be possible to re-sensitize cancer cells to apoptosis, thereby inhibiting tumor growth and enhancing the efficacy of cancer therapies. Thus, elucidating the importance of Cyt C in the context of cancer metabolism and apoptosis provides a promising avenue for developing targeted therapies and improving patient outcomes in cancer treatment.

## 10 Conclusion

Cytochrome *c* (Cyt C) is crucial in mitochondrial respiration and apoptosis. When the mitochondrial outer membrane is disrupted, Cyt C is released into the cytoplasm and can enter the bloodstream, where it influences apoptosis. In cancer, apoptosis is often inhibited, making Cyt C a valuable target for therapy. Elevated serum levels of Cyt C indicate increased apoptosis, while decreased levels suggest its inhibition. Measuring Cyt C in serum can track chemotherapy effectiveness and provide prognostic insights. Sensitive biosensors for quantifying Cyt C levels have been developed, and exogenous Cyt C delivery can effectively induce cancer cell apoptosis. The development of advanced delivery systems and improvements in Cyt C’s stability further enhance its therapeutic potential. Collectively, these factors underscore Cyt C’s potential as a powerful tool in both the direct treatment of cancer and the optimization of therapeutic strategies, making it a promising candidate for future cancer therapies.

## 11 Declarations

**Conflict of Interest:** The author has no conflict of interest to declare, financial or otherwise.

**Ethical Approval:** Not Applicable.

## Declaration of competing interest

The authors declare that they have no known competing financial interests or personal relationships that could have appeared to influence the work reported in this paper.
